# THB1, a putative transmembrane protein that causes hybrid breakdown in rice

**DOI:** 10.1270/jsbbs.23065

**Published:** 2024-06-13

**Authors:** Tae Wakabayashi, Kiyoaki Kato

**Affiliations:** 1 Department of Agro-Environmental Science, Obihiro University of Agriculture and Veterinary Medicine, Nishi 2-11 Inada, Obihiro, Hokkaido 080-8555, Japan

**Keywords:** reproductive isolation, hybrid breakdown, rice, putative transmembrane protein, haplotype, derived cleaved amplified polymorphic sequence marker

## Abstract

Hybrid breakdown is a post-zygotic reproductive isolation that hinders genetic exchange between species or populations in both animals and plants. Two complementary recessive genes, *temperature sensitive hybrid breakdown1* (*thb1*) and *thb2*, cause hybrid breakdown in rice (*Oryza sativa*). The present study delimited the *THB1* locus to a 9.1-kb sequence, containing a single gene encoding a putative transmembrane protein with unknown functions. Haplotype analysis of *THB1* in the two core collections of 119 accessions revealed that these accessions were divided into 22 haplotypes. A test cross with *thb2* carrier showed that haplotype2 (H2) was assigned to *thb1* and was restricted to *temperate japonica*. A nonsynonymous nucleotide polymorphism (SNP) specific to H2 was identified as a causal mutation in *thb1*. A test cross with *thb1* carrier indicated that six accessions, including *temperate japonica*, *tropical japonica*, and *indica*, carried *thb2*. These results suggest that *thb1* has recently evolved in *temperate japonica*, whereas *thb2* arose in an ancient *japonica* and introgressed into the present three subgroups. Furthermore, we developed a derived cleaved amplified polymorphic sequence (dCAPS) marker to detect causal SNP in *THB1*. Our findings provide new insights into reproductive isolation and may benefit rice breeding.

## Introduction

Reproductive isolation hinders genetic exchange between species and populations of both plants and animals. Therefore, it is considered fundamental for speciation (Coyne and Orr 2004). Generally, reproductive isolation in plants can be classified into pre- and post-pollination, according to the developmental stage during which it occurs. Pre-pollination isolation, such as habitat divergence, temporal isolation, pollinator isolation, and mating system divergence, usually functions more effectively than post-pollination isolation (Coyne and Orr 2004). Post-pollination reproductive isolation can divide into pre-zygotic and post-zygotic isolation. Post-zygotic reproductive isolation is common across the plant and animal kingdoms (Dobzhansky 1970, Grant 1971, Stebbins 1958). Hybrids that experience postzygotic reproductive isolation are usually aborted or arrested at different stages or generations after fertilization. Based on the developmental stage at which isolation occurs and the symptoms it displays, post-zygotic reproductive isolation can be termed as hybrid inviability, hybrid weakness, hybrid necrosis, and hybrid sterility, which are observed in F_1_ hybrids, and hybrid breakdown in F_2_ and backcross generations.

Hybrid breakdown facilitates speciation by restricting gene flow between diverging taxa in both animal and plant species (Stebbins 1958) and reduces selection efficiency in crossbreeding. One of the fundamental theories on the mechanism of post-zygotic isolation is the Bateson–Dobzhansky–Muller (BDM) model (Bateson 1909, Dobzhansky 1937, Muller 1942). BDM model postulates that deleterious interactions between two or more genes derived from different species or populations cause post-zygotic isolation. Two molecular mechanisms underlying the BDM-type hybrid breakdown have been reported in plants: an elevated autoimmune response associated with the nucleotide binding site-leucine-rich repeat (NBS-LRR) genes (Alcázar *et al.* 2009, Bomblies *et al.* 2007, Yamamoto *et al.* 2010) and reciprocal silencing or loss of duplicated genes (Bikard *et al.* 2009, Kubo *et al.* 2022, Vlad *et al.* 2010). However, in many cases, the causal genes remain unknown. Elucidation of the mechanisms of hybrid breakdown is important not only to understand speciation and evolution, but also to overcome these barriers for crop breeding.

In our previous study, two complementary recessive genes, *temperature sensitive hybrid breakdown1* (*thb1*) and *thb2*, that cause hybrid breakdown were serendipitously found in closely related *temperate* *japonica* cultivars of rice (*Oryza sativa*), ‘Yukihikari’ and ‘Kirara397’, respectively (Yoneya *et al.* 2021). In this study, we identified a single nonsynonymous nucleotide polymorphism (SNP) as the causal mutation in *THB1* that encodes a putative transmembrane protein with unknown functions. We also demonstrated that *thb1* is restricted to *temperate japonica*, whereas *thb2* is distributed not only in *temperate japonica* but also in *tropical japonica* and *indica*. Furthermore, we developed a derived cleaved amplified polymorphic sequence (dCAPS) marker to determine the causal SNP genotype of *THB1*. Our findings provide new insights into reproductive isolation and may benefit rice breeding.

## Materials and Methods

### Plant materials

The rice plants cultivars ‘Yukihikari’ and ‘Kirara397’, and a chromosome segment substitution line YK3CSSL-6.1 (Kato and Hirayama 2021, Yoneya *et al.* 2021) were used as parental controls for mapping study. For the fine-scale mapping of *THB1*, a total of 3,754 F_2_ plants of a cross between ‘Kirara397’ and YK3CSSL-6.1 and self-pollinated F_3_ progeny were used. To characterize the distributions of *thb1* and *thb2*, nine accessions in a Core Collection of Japanese rice landraces (JRC) of the NARO GenBank Project (Ebana *et al.* 2008), ‘Mansaku’ (JRC22/JP6735), ‘Shinyamadaho 2’ (JRC37/JP6962), ‘Akage’ (JP14994, JP5301), ‘Sensho’ (JRC04/JP4386), ‘Karahoushi’ (JRC44/JP10788), ‘Touboshi’ (JRC42/JP10772), and ‘Akamai’ (JRC43/JP4744, JRC21/JP9694) were used.

### InDel and SNP marker analyses

Two Insertion-deletion (InDel) markers, YK3InDel06-845078_2 and YK3InDel06-646646, were used (Yoneya *et al.* 2021). In the present study, nine SNPs were converted into a cleaved amplified polymorphic sequence (CAPS) marker and eight dCAPS markers based on our previous research (Takano *et al.* 2014) and using the web-based free software program dCAPS Finder 2.0 (Neff *et al.* 2002) between YK3InDel06-845078_2 and YK3InDel06-646646. Appropriate PCR primer sets flanking each target SNP were designed using Primer 3.0 (version 0.4.0) software (Supplemental Table 1). Young leaves of the parents and the mapping population were collected to extract DNA and subjected to marker analyses as described by Yoneya *et al.* (2021).

### Sanger sequencing

Repeat number of T on chr06: 799,424..799,433 (IRGSP-1.0) was determined using Sanger sequencing and designated as YK3InDel-799424. Target DNA fragment was amplified using forward primer 5ʹ-TTGGGGTACCTTGAAGATGTG-3ʹ and reverse primer 5ʹ-TCCCCTCTCTAGCTCCTCTTG-3ʹ. PCR reaction were performed using a thermocycler GeneAtlas G (Astec, Fukuoka, Japan) for a total volume of 20 μL, including 2 μL 10× Ex taq buffer (Takara, Shiga, Japan), 1.6 μL dNTPs (2 mM), 0.2 μL Ex taq (5 U/μL), 0.8 μL primer each (10 μM), 2 μL DNA template (30 ng/μL), and 12.6 μL of DNase-free water. The PCR amplification program was as follows: 2 min at 98°C, 35 cycles of 10 s denaturation at 98°C, 30 s annealing at 60°C, and 30 s extension at 72°C. Purified PCR products were subjected to Sanger sequencing (Eurofins Genomics, Tokyo, Japan) to detect the number of T of the targeted bases.

### Growth condition

For genetic mapping and test cross studies, the seeds of F_2_ populations, F_3_ lines, and parental cultivars were sterilized with 0.2% (w/v) benlate solution for 1 d and germinated in reverse osmosis water for 2 d in the dark at 30°C, planted in a soil-filled cell plug tray (cell count, 8 × 16; tray size, 52 × 25 cm^2^; cell size, 3 × 3 × 4.4 cm^3^). Each cell plug tray was placed in a plastic container (53 × 34.8 × 15.6 cm^3^), and 3–10 cm of water was maintained depending on the plant size. To screen the recombinant F_2_ plants between YK3InDel06-845078_2 and YK3InDel06-646646 (Yoneya *et al.* 2021), F_2_ plants and parental control, ‘Yukihikari’, ‘Kirara397’, and YK3CSSL-6.1, were planted four times during 2020; February 14, April 3, April 24, and June 29. For progeny tests to determine *THB1* genotypes, 23–56 F_3_ plants derived from each recombinant F_2_ plant were planted with parental control three times during 2020; August 17, October 16, and November 12. F_2_ populations for test cross study were planted on October 16, 2022. These plant materials were grown in a greenhouse at Obihiro University of Agriculture and Veterinary Medicine (OUAVM; 42°52ʹN, 143°9ʹE), with temperature maintained over 20°C from November to May. From the end of May to October, the plants were grown in a glasshouse, with the daily mean temperature maintained less than 25°C.

The F_1_ plants were grown under two conditions in the test cross study. First, F_1_ plants were grown in a greenhouse at OUAVM and grown at temperatures >25°C from December 2021 to April 2022 under natural day-length conditions. Second, F_1_ plants were grown at temperatures >25°C in a greenhouse from April to May and >20°C in a glasshouse at OUAVM from the end of May to October 2022 under natural day-length conditions. In both experiments, seeds were sterilized and planted as previously described. Four-week-old F_1_ plants were transplanted into plastic pots filled with 2L soil compost containing 1.2 g of N, P_2_O_5_, and K_2_O each. Spikelet fertility (percentage of filled spikelets per total number of spikelets) was assessed using five culms per plant, for five individuals from each cross.

### Gene expression analysis

The plants were grown in a growth cabinet (Biotron, NK Systems, Japan) under 25°C with a 16 h-light (350 μmol/m^2^/s)/8 h-dark photoperiod in water. One week after germination, the shoots and roots of five seedlings were pooled as biological replicates. The experiments were performed using three biological replicates. Total RNA was extracted from each tissue sample using the RNAsuisui-S reagent (Rizo Co., Tsukuba, Japan). Reverse transcription-polymerase chain reaction (RT-PCR) was performed using a PrimeScript™ II first strand cDNA Synthesis Kit (Takara Bio, Kyoto, Japan). DNase digestion was performed using DNase I (Nippon Gene, Tokyo, Japan).

The transcriptional variants of *THB1* were amplified using the following two primer sets: forward primer 5ʹ-CCATGAGATGTCCATGTACCAG-3ʹ and reverse primer 5ʹ-GAGGATTCCAATCGCCCATGA-3ʹ (thb1-1), and forward primer 5ʹ-CAAGAGCCACAGGTGGAGAG-3ʹ and reverse primer 5ʹ-TAGCACCAATGCAGCGTACA-3ʹ (thb1-2). Quantitative RT-PCR was performed using a 7300 Real-Time PCR System (Applied Biosystems, USA) and SYBR Premix Ex Taq II (Takara Bio). *OsUBQ1* (Os03g0234200) was used as the reference gene (Yamamoto *et al.* 2010). The expression levels in each sample were calculated using three technical replicates.

### Haplotype network analysis

Genome-wide variation data from TASUKE+ (Kumagai *et al.* 2019, https://tasuke.dna.affrc.go.jp) for the rice core collection of the World Rice Core Collection (WRC) of the NARO GenBank Project (Kojima *et al.* 2005) and the JRC of the NARO GenBank Project (Ebana *et al.* 2008) were used to identify genetic polymorphisms in *THB1* region. To construct a Templeton, Crandall, and Sing haplotype network (Templeton *et al.* 1992), Population Analysis with Reticulate Trees software (version 1.7, PopART; https://popart.maths.otago.ac.nz/download/) was used.

### Phylogenetic analysis

A phylogenetic tree was constructed by Molecular Evolutionary Genetics Analysis (version 11, MEGA 11; Tamura *et al.* 2021) using the neighbor-joining method (Saitou and Nei 1987). The bootstrap consensus tree inferred from 1000 replicates was used to represent the evolutionary history of the taxa analyzed (Felsenstein 1985). The tree was drawn to scale, keeping the units of measurement of branch lengths same as those of the evolutionary distances used to infer the phylogenetic tree. Evolutionary distances were computed using the Kimura 2-parameter method (Kimura 1980). All positions containing gaps or missing data were eliminated from the dataset (complete deletion option).

## Results

### Fine-scale mapping of *THB1* locus

**Fig. 1.**
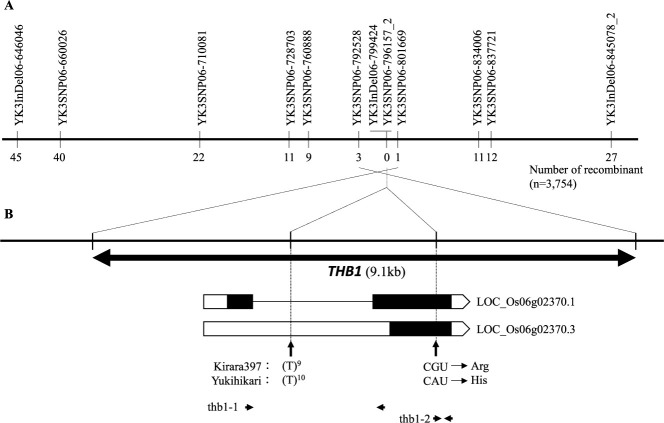
Map-based cloning of *THB1*. (A) High-resolution linkage map of *THB1* using 72 recombinants between YK3InDel06-845078_2 and YK3InDel06-646646 from 3,754 F_2_ individuals in the present study. (B) Structures of alternative splicing variants of LOC_Os06g02370. The full-length protein encoded by the longest transcript variant (LOC_Os06g02370.1) is 584 amino acids in length and was taken as reference for intron/exon numbering. LOC_Os06g02370.3 was generated by miss-splicing of intron resulting in less intron and changing the position of start codon, which causes lack of 1^st^ exon and reducing 5ʹ site of 2^nd^ exon (332 amino acids). White boxes represent 5ʹ-UTR; white pentagons represent 3ʹ-UTR; black boxes represent exon; and horizontal lines between them represent introns. The vertical arrows indicate the polymorphisms between ‘Yukihikari’ and ‘Kirara397’ in LOC_Os06g02370. Two sets of short horizontal arrows represent the positions of primers, thb1-1 and thb1-2, for expression analyses.

In our previous study, *THB1* was mapped between two InDel markers, YK3InDel06-845078_2 and YK3InDel06-646646 (Yoneya *et al.* 2021). To narrow down the candidate region of *THB1*, 3,754 F_2_ plants were screened for recombination between these two InDel markers. Seventy-two recombinant F_2_ plants were selected and their genotypes for *THB1* were evaluated based on the segregation patterns of plant growth in the F_3_ progeny. Weak phenotypes were discriminated based on visual observations, including leaf length, width, and color (Yoneya *et al.* 2021). Nine SNP markers were genotyped from these 72 individuals. Among them, three recombinants were detected between YK3SNP06-792528 and *THB1*. On the other hand, a single F_2_ plant (Plant ID: K19-24-2-3) was screened for a recombinant chromosome between YK3SNP06-801669 and *THB1*. Next, we determined the K19-24-2-3 genotype at YK3InDel-799424 using Sanger sequencing. The *THB1* gene was co-segregated with YK3InDel-799424 and YK3SNP06-796157_2 and eventually mapped at a 9.1-kb interval between YK3SNP06-792528 and YK3SNP06-801669 on the short arm of chromosome 6 (Fig. 1A). Based on our re-sequencing data, no other polymorphisms were detected in the *THB1* locus (9.1 kb) between the parent cultivars (Takano *et al.* 2014).

YK3SNP06-792528 is located 2,803 bp downstream of LOC_Os06g02370. YK3SNP06-801669 is located 1,856 bp upstream of LOC_Os06g02370. Thus, the region delimited by fine mapping contained only LOC_Os06g02370, according to *O. sativa* ssp. *japonica* cv. ‘Nipponbare’ (annotated by Rice Genome Annotation Project, http://rice.uga.edu/) (Fig. 1B). Two splicing variants, LOC_Os06g02370.1 (584 amino acids) and LOC_Os06g02370.3 (332 amino acids), were annotated as LOC_Os06g02370. YK3InDel-799424 was assigned to the intron of LOC_Os06g02370.1 and 5ʹ-UTR of LOC_Os06g02370.3. YK3SNP06-796157_2 was located at the exon of both transcript variants and had replaced arginine in ‘Kirara397’ with histidine in ‘Yukihikari’.

### Sequence analysis of LOC_Os06g02370

**Fig. 2.**
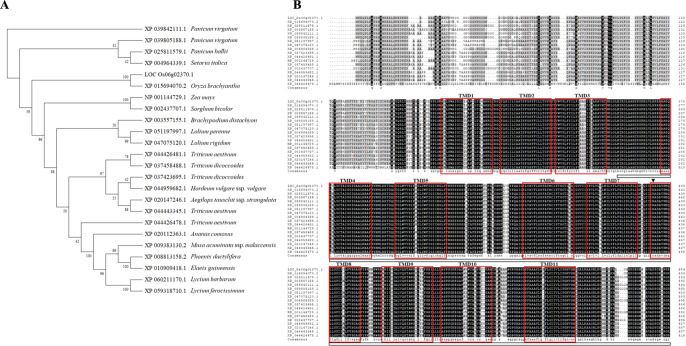
Phylogenic tree and multiple sequences alignment of THB1 (LOC_Os06g02370.1) with its highly similar protein sequences. Amino acid sequences of XP_015642768.1 (THB1, LOC_Os06g02370.1) from *Oryza sativa* ssp. *japonica* group; XP_015694070.2 from *Oryza brachyantha*; XP_025811579.1 from *Panicum hallii*; XP_003557155.1 from *Brachypodium distachyon*; XP_039842111.1 and XP_039805188.1 from *P. virgatum*; XP_051197997.1 from *Lolium perenne*; XP_047075120.1 from *L. rigidum*; XP_004964339.1 from *Setaria italica*; XP_037423695.1 and XP_037458488.1 from *Triticum dicoccoides*; XP_044426481.1, XP_044443345.1, and XP_044426478.1 from *T. aestivum*; NP_001144729.1 from *Zea mays*; XP_002437707.1 from *Sorghum bicolor*; XP_044959682.1 from *Hordeum vulgare* ssp. *vulgare*, XP_020147246.1 from *Aegilops tauschii* ssp. *strangulate*; XP_020112363.1 from *Ananas comosus*; XP_008813158.2 from *Phoenix dactylifera*; XP_010909418.1 from *Elaeis guineensis*; XP_009383130.2 from *Musa acuminata* ssp. *malaccensis*; XP_060211170.1 from *Lycium barbarum*; and XP_059318710.1 from *L. ferocissimum* were compared. (A) Phylogenic tree of THB1-like proteins. (B) Amino acid sequence alignment of 17 THB1-like proteins (amino acid identity >87.1% to LOC_Os06g02370.1). The amino acid sequence of LOC_Os06g02370.3 is indicated by white box. Predicted transmembrane domains (TMD) are indicated with red boxes. Identical and similar amino acid residues are shaded in black and gray, respectively. A nonsynonymous mutation site is indicated using a triangle.

To obtain more information about LOC_Os06g02370, bioinformatics analysis was performed using the amino acid sequence of LOC_Os06g02370.1. High similarity of protein sequences (amino acid identity >50% to LOC_Os06g02370.1) was selected for bioinformatics analysis. Twenty-three LOC_Os06g02370.1-like proteins (amino acid identity >50.4% to LOC_Os06g02370.1) were identified in plant; namely, XP_025811579.1 from *Panicum hallii*; XP_039842111.1 and XP_039805188.1 from *P. virgatum*; XP_004964339.1 from *Setaria italica*; NP_001144729.1 from *Zea mays*; XP_002437707.1 from *Sorghum bicolor*; XP_015694070.2 from *Oryza brachyantha*; XP_003557155.1 from *Brachypodium distachyon*; XP_051197997.1 from *Lolium perenne*; XP_047075120.1 from *Lolium rigidum*; XP_044959682.1 from *Hordeum vulgare* ssp. *vulgare*; XP_037423695.1 and XP_037458488.1 from *Triticum dicoccoides*; XP_044426481.1, XP_044443345.1, and XP_044426478.1 from *Triticum aestivum*; XP_020147246.1 from *Aegilops tauschii* ssp. *strangulate*; XP_020112363.1 from *Ananas comosus*; XP_008813158.2 from *Phoenix dactylifera*; XP_010909418.1 from *Elaeis guineensis*; XP_009383130.2 from *Musa acuminata* ssp. *malaccensis*; XP_060211170.1 from *Lycium barbarum*; and XP_059318710.1 from *Lycium ferocissimum*. Based on these proteins, an evolutionary tree was constructed (Fig. 2A).

Subsequently, multiple sequence alignments were performed among LOC_Os06g02370.1 and 17 LOC_Os06g02370.1-like proteins (amino acid identity >87.1% to LOC_Os06g02370.1) (Fig. 2B). Conserved domain analysis using SOSUI 1.11 revealed that LOC_Os06g02370.1 contains eleven transmembrane domains (TMD), which is highly conserved region among monocot 17 LOC_Os06g02370.1-like proteins (Fig. 2B). The R426H mutation in LOC_Os06g02370.1 was localized in TMD 8.

Phylogenetic comparisons of these proteins showed that they are highly conserved, and exhibit relatively close genetic relationships. However, none of the selected protein sequences have been studied in detail, and their functions are unknown; therefore, we concluded that *THB1* is a putative transmembrane protein with unknown function.

### Phylogenic and haplotype network analyses at LOC_Os06g02370 among JRC and WRC

**Fig. 3.**
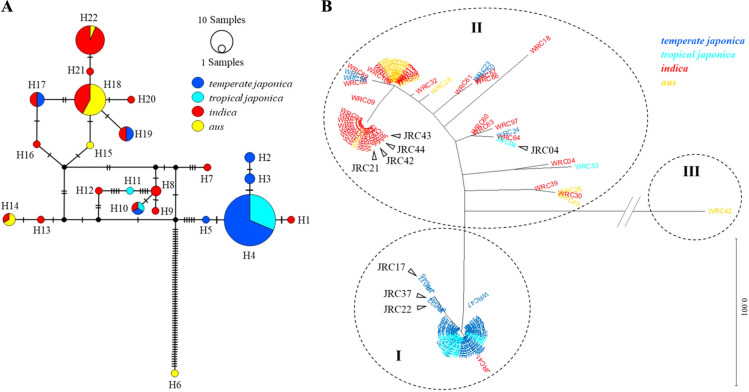
Phylogenic and network analysis of *THB1* gene. (A) Haplotype network of the *THB1* in WRC and JRC. The size of each circle is proportional to the accession numbers encompassed, and different colors indicate the subgroups. The number of hatch marks reflects the number of nucleotide differences detected between haplotypes. (B) Phylogenic tree of *THB1* based on nucleotide sequences of WRC and JRC. *Arrowheads* indicate the JRC ID used in test-cross experiments.

Haplotype network and phylogenic analyses were conducted to visualize the genetic relationships between the LOC_Os06g02370 region (6503 bp, chr06: 796,310..802,812), covering all the identified exons, introns, and UTRs and the promoter region (2,000 bp), among 69 WRCs and 50 JRCs. In total, 123 polymorphisms, including 96 SNPs and 27 InDels, were detected in the 119 accessions. Nineteen haplotypes were identified in the WRC and six haplotypes were identified in the JRC. Three haplotypes (H4, H10 and H22) were shared between both collections (Fig. 3A). Twenty-two haplotype variants were identified. Two accessions, ‘Akage’ (JRC17) and ‘Fukoku’ (JRC46/JP14924) were classified to haplotype2 (H2), which was identical to ‘Yukihikari’. Because the seed of ‘Akage’ (JRC17) was not available from NARO GenBank, two accessions named ‘Akage’ (JP5301 and JP14994) were used to confirm the genotype at YK3InDel-799424 and YK3SNP06-796157_2. All results confirmed that two accessions (‘Akage’) were identical to H2. Among 22 haplotypes, only H2 had a nonsynonymous SNP at YK3SNP06-796157_2.

To determine the genetic association of the LOC_Os06g02370 region among the classified subpopulations, we investigated the association of three major haplotypes (H4, H18, and H22) carried by more than ten accessions. H18 and H22 were closely related and specific to *indica* and *aus* (Fig. 3A). These two haplotypes were distantly associated with *temperate japonica*- and *tropical japonica*-specific H4, indicating different variations in LOC_Os06g02370 among subpopulations (Fig. 3A).

To understand the reticulated relationship between the LOC_Os06g02370 haplotypes, a phylogenetic analysis was conducted using sequence data from TASUKE+ for the WRC and JRC (Fig. 3B). One hundred and nineteen sequences were classified into three clusters: Clusters I–III. Cluster I predominantly comprised *temperate japonica* and *tropical japonica*. Clusters II and III were predominantly *indica* and *aus*. Taken together with the haplotype network and phylogenetic analyses of the LOC_Os06g02370 region, we concluded that H2 was derived from H4 via H3 in *temperate japonica*.

### Spikelet fertilities of F_1_ hybrids in test cross study

**Fig. 4.**
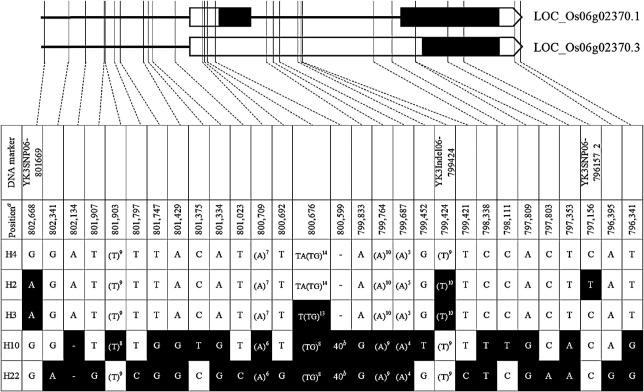
Gene structure and sequence alignment of the *THB1* region. H4 is reference haplotype being carried by 51 accessions from 119 accessions from JRC and WRC. White cells indicate the same nucleotide as that of the reference haplotype. Black cells indicate variants. *a* indicates physical position on chromosome 6 (Nipponbare IRGSP-1.0 reference genome). *b* indicates 40-bp indel polymorphism of AAGCTACACAAACACAACGGAATGGTGAAGGTATAGAGAG.

To address the distribution of *thb1* and *thb2*, *thb2* carrier ‘Kirara397’ and *thb1* carrier ‘Yukihikari’ were test-crossed with nine accessions (Fig. 4, Table 1). Hybrid sterility is commonly observed in inter-subspecific crosses, leading to reduced fitness and segregation distortion in the offspring (Chen *et al.* 2008, Kubo *et al.* 2016, Li *et al.* 2017, Long *et al.* 2008, Shen *et al.* 2017, Yang *et al.* 2012). We characterized the spikelet fertility of the F_1_ hybrids and classified them into three groups: Group A, >61%; Group B, >21% and <60%; and Group C, <20%. F_1_ spikelet fertility of the crosses between testers, four *temperate japonica* accessions, and a *tropical japonica* accession was consistently maintained at >61% and classified as group A. The F_1_ spikelet fertility of the crosses between two testers and two *indica* accessions (‘Akamai’) varied from 2.1% to 65.3%. In these four cross combinations, F_1_ spikelet fertility in 2022 (summer) was higher than that in 2021 (winter) suggesting the F_1_ sterility of the crosses with these two accessions (‘Akamai’) were affected by the environmental conditions. In addition, F_1_ spikelet fertility of the crosses between ‘Yukihikari’ and two *indica* accessions (‘Akamai’) was consistently lower than that of the crosses between ‘Kirara397’ and two *indica* accessions (‘Akamai’), and were classified as severe sterility (group C). These results suggested that the conditional F_1_ sterility was diverged genetically between ‘Kirara397’ and ‘Yukihikari’.

F_1_ spikelet fertility of the crosses between testers and two *indica* accessions, ‘Karahoushi’ and ‘Touboshi’, ranged from 0.4% to 24.6%, and was classified as group C. F_1_ fertility of the crosses between ‘Kirara397’ and the two *indica* accessions in 2022 (summer) was higher than that in 2021 (winter), supporting that F_1_ fertility of these cross combinations was affected by environmental conditions. F_1_ fertility of the crosses between ‘Yukihikari’ and these two *indica* accessions ranged from 1.2% to 8.8%. Under both conditions, F_1_ fertilities of ‘Yukihikari’/‘Touboshi’ were consistently higher than those of ‘Yukihikari’/‘Karahoushi’.

### Identification of *THB1* haplotype responsible for hybrid breakdown

We assessed the growth habits of F_2_ plants derived from the crosses between *thb2* carrier, ‘Kirara397’, and nine accessions (Fig. 4, Table 1). Thereafter, we calculated the segregation ratio of normal versus weak plants in each F_2_ population to determine whether the weak plants were genetically regulated by the two complementary recessive genes. The segregations ratios of normal to weak plants were 465:27 in F_2_ population of ‘Kirara397’/‘Akage’ (JP5301) and 448:36 in F_2_ population of ‘Kirara397’/‘Akage’ (JP14994). These correspond to a ratio of 15:1 (χ^2^ = 0.36, *p* = 0.55; χ^2^ = 1.17, *p* = 0.28) for two complementary recessive genes.

Complementary interactions were identified between *thb2* and *thb1*(H2) from landrace ‘Akage’ (JP5301, JP14994), as shown in a cross with ‘Yukihikari’ (Yoneya *et al.* 2021). No interactions were detected between *thb2* and H3 or H10 and H22. Finally, an H2-specific nonsynonymous SNP at YK3SNP06-796157_2 was identified as a causal mutation in *thb1* (Fig. 4).

### Distribution of *thb2*

We evaluated the segregation ratios of normal versus weak plants in each F_2_ population of the crosses between ‘Yukihikari’, the *thb1* carrier, and the nine target accessions to assess whether the weak plants were genetically regulated by the complementary recessive genes. The segregation ratios of normal to weak plants were 427:33 in F_2_ population of ‘Yukihikari’/‘Mansaku’, 415:28 in F_2_ population of ‘Yukihikari’/‘Shinyamadaho 2’, 378:24 in F_2_ population of ‘Yukihikari’/‘Sensho’, and 272:18 in F_2_ population of ‘Yukihikari’/‘Touboshi’. This corresponds to a 15:1 ratio for the two complementary recessive genes. In contrast, slight segregation distortion was observed in the crosses, ‘Yukihikari’/‘Akamai’ (JRC21/JP9694) and ‘Yukihikari’/‘Akamai’ (JRC43/JP4744). The segregation ratios of normal to weak plants were 408:15 in F_2_ population of ‘Yukihikari’/‘Akamai’ (JRC21/JP9694) and 415:15 in F_2_ population of ‘Yukihikari’/‘Akamai’ (JRC43/JP4744), indicating slight deficiency of double recessive genotype in both populations.

The present results demonstrated that *thb2* was distributed in not only *temperate* *japonica* (‘Mansaku’ and ‘Shinyamadaho 2’) but also *tropical japonica* (‘Shensho’) and *indica* (‘Akamai’ and ‘Touboshi’).

### Gene expression analysis of LOC_Os06g02370

**Fig. 5.**
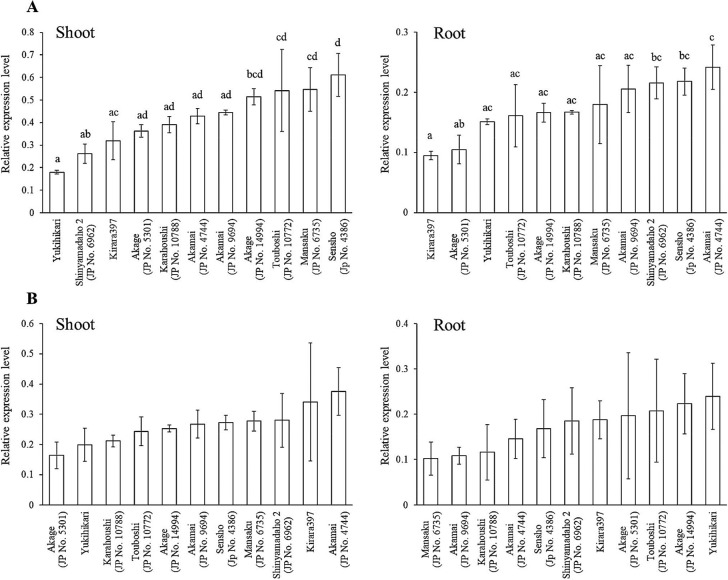
Relative expression level of *THB1* in nine accessions in 10 days after germination. (A) Relative expression levels of the LOC_Os06g02370.1 gene using primer set of thb1-1 were detected using quantitative RT-PCR. (B) Combined relative expression level of LOC_Os06g02370.1 and LOC_Os06g02370.3 using the primer set of thb1-2 were detected using quantitative RT-PCR. All data were normalized to the expression level of *OsUBQ1* (Os03g0234200) (Yamamoto *et al.* 2010); *error bars* are SD for three independent experiments. Different letters indicate significant difference at *p* < 0.05 (Tukey’s test).

To address the possible association between the expression level of *THB1* and hybrid breakdown, the gene expression levels of LOC_Os06g02370 at the seedling stage of 11 accessions were analyzed using quantitative RT-PCR. The expression levels of LOC_Os06g02370.1 using primer thb1-1 varied among accessions in the shoots and roots (Fig. 5). In shoots, the highest expression was observed in ‘Sensho’ (JRC04/JP4386), and the lowest expression was observed in ‘Yukihikari’. In roots, the highest expression was observed in ‘Akamai’ (JRC43/JP4744), and the lowest expression was observed in ‘Kirara397’. In both tissues, significant differences were noted among *japonica* accessions, but not among *indica* accessions. In contrast, similar amounts of combined expression of LOC_Os06g02370.1 and LOC_Os06g02370.3 using primer thb1-2 were observed among all tested accessions in both tissues. Although ‘Yukihikari’ and two accessions of ‘Akage’ carry H2, the expression levels of LOC_Os06g02370.1 were independent of the haplotype. Collectively, these results exclude the possibility that the expression level of *THB1* is associated with hybrid breakdown.

## Discussion

### *THB1* encodes a novel putative transmembrane protein

We found that *THB1* is a novel causal gene that causes hybrid breakdown. Furthermore, this study revealed that *THB1*-like genes are distributed as a single copy per genome in each plant, as in the case of rice. It may play an essential role in the development and survival of plants. However, to date, no evidence has revealed the function of THB1 and its ortholog in the vegetative and reproductive development of either rice or other plants. Therefore, it would be interesting to determine the functions of THB1 and its orthologs. THB1 contains multiple putative TMDs without predicted signal peptides that are conserved across orthologs. Further studies are required to clarify the subcellular localization of THB1 and elucidate its function.

The present study clarified that a single amino acid substitution of R426H in LOC_Os06g02370.1, and R172H in LOC_Os06g02370.3 in the well-conserved putative transmembrane domain is the causal mutation for hybrid breakdown. To date, two molecular mechanisms of hybrid breakdown in plants have been reported: reciprocal silencing or loss of duplicated genes (Kubo *et al.* 2022, Vlad *et al.* 2010) and the elevated autoimmune response associated with the NBS-LRR gene (Alcázar *et al.* 2009, Yamamoto *et al.* 2010). In rice, the loss of duplicated Esa-associated factor 6 (EAF6) genes on chromosomes 1 and 12 causes hybrid breakdown (Kubo *et al.* 2022). However, the present study showed that the rice genome does not contain duplicated *THB1*. In contrast, *hybrid breakdown2* (*hbd2*) and *hbd3* encode casein kinase I (CKI1) and NBS-LRR, respectively (Yamamoto *et al.* 2010). *hbd2-CKI1* allele gains its deleterious function that causes a weak phenotype by changing one amino acid, with hybrid breakdown attributed to an elevated autoimmune response. Yamamoto *et al.* (2010) suggested that *hbd2*-CKI1 acts like Avr protein or a protein disturbed by Avr protein, with *hbd3*-NBS-LRR(s) recognizing *hbd2*-CKI1 directly or indirectly to trigger the immune response signal. As in the case of gene combinations of *hwj1* and *hwj2*, overactivated immune responses may trigger temperature-sensitive hybrid breakdown (Soe *et al.* 2022). In general, ambient temperature is a key environmental factor affecting the strength of plant immune responses, and pathogen effector-triggered immunity (ETI) is preferentially activated in plants at low temperatures (Cheng *et al.* 2013), indicating that the genes involved in ETI are more likely to be recruited for establishing low temperature-induced hybrid breakdown. The present study demonstrated that THB1 (R426/172H) may act like Avr or a protein disturbed by Avr as in case of *hbd2*-*hbd3* system (Yamamoto *et al.* 2010). Now, we are challenging the map-based cloning of *THB2* to clarify the molecular mechanism whether the ETI system or a novel molecular mechanism contributes to the present low temperature-dependent hybrid breakdown.

### Evolutionally history of *thb1*(H2) and *thb2*

Haplotype network analysis of *THB1* locus among 119 accessions in the JRC (Ebana *et al.* 2008) and WRC (Kojima *et al.* 2005) revealed that *thb1*(H2) was originated from the major haplotype of *Thb1*(H4) via *Thb1*(H3). Notably, both *thb1*(H2) and *Thb1*(H3) were restricted to *temperate japonica* of the JRC, although *Thb1*(H4) was distributed in *temperate* *japonica* and *tropical japonica* in both collections. These results suggest that *thb1* has recently evolved in *temperate japonica*. The present molecular marker analysis using YK3SNP06-796157_2 and Sanger sequence at YK3InDel-799424 confirmed that two accessions named ‘Akage’ (JP5301 and JP14994) carry *thb1*(H2). ‘Akage’ is a forerunner landrace of the rice cultivars in Hokkaido, followed by its role as parents in pure-line selection and modern cross breeding. ‘Fukoku’ and ‘Yukihikari’ were developed from the progeny of ‘Akage’. Therefore, *thb1*(H2) may be inherited by ‘Fukoku’ and ‘Yukihikari’ from ‘Akage’. ‘Akage’ has originated from Akita Prefecture, northern Japan. It is necessary to address the geographical distribution of *thb1*(H2) using more accessions to clarify the evolutionary history of *THB1* locus, particularly if *thb1*(H2) originated from *Thb1*(H3) in the Akita Prefecture.

The present test cross study revealed that *thb2* was distributed across three subgroups: *temperate* *japonica*, *tropical japonica*, and *indica*. Previous studies have shown that the domestication of Asian cultivated rice was from a single origin (Huang *et al.* 2012, Molina *et al.* 2011), which proposes the cycles of the introgression hypothesis. Based on this theory, wild rice was first domesticated as the ancient *japonica*. One part of ancient *japonica* hybridized with local wild rice in South Asia to form the *indica* subgroup. Another ancient *japonica* group was domesticated into the modern *japonica* group. The *japonica* subgroup is widely distributed across East Asia. It is divided into the *temperate japonica* and *tropical japonica* subgroups. The results presented here suggest that *thb2* arose in an ancient *japonica* and introgressed into the other extant subgroups. In future studies, molecular cloning of *THB2* locus, haplotype analysis, and distribution of *thb2* using a large collection of Asian rice will clarify the evolutionary history of *thb2*.

### Divergence of hybrid sterility between ‘Kirara397’ and ‘Yukihikari’

In the present test cross study, the spikelet fertility of F_1_ hybrids of *temperate* *japonica*/*temperate* *japonica* and *temperate* *japonica*/*tropical japonica* crosses was consistently greater than 61%, whereas those of *japonica*/*indica* crosses were varied from almost completely sterile to half sterile among the cross combinations, without hybrid weakness. Among eight *temperate* *japonica*/*indica* crosses, the spikelet fertility of F_1_ plants crossed with ‘Yukihikari’ was consistently lower than that of F_1_ plants crossed with ‘Kirara397’. Hybrid sterility is a complex quantitative trait controlled by multiple loci. Maekawa *et al.* (1998) reported that ‘Yukihikari’ carried different hybrid sterility genes from another *japonica* cultivar ‘T-65’ in *japonica*/*indica* hybrids. However, to date, no genetic analysis has been performed to differentiate the hybrid sterility loci in ‘Yukihikari’. More than 22 loci responsible for hybrid sterility in crosses between different subgroups have been identified using genetic mapping study (*reviewed by* Zhang *et al.* 2022). Further studies are required to confirm which hybrid sterility genes contributed to varied sterility between ‘Yukihikari’/*indica* and ‘Kirara397’/*indica*. This will clarify the possibility that the differentiation in combinations of distinct hybrid sterility loci diverged between ‘Yukihikari’ and ‘Kirara397’.

In the present study, the double recessive genotype, *thb1 thb2*, was slightly deficient in the F_2_ populations of the crosses ‘Yukihikari’/‘Akamai’ (JRC21/JP9694 and JRC43/JP4744), resulting slight segregation distortion. To date, two genetic models have been proposed to explain hybrid sterility. One model is the one-locus sporo-gametophytic interaction model (Kitamura 1962), and was followed by *S5*, *S7*, *Sa*, *Sc*, and *HSA1* (Chen *et al.* 2008, Kubo *et al.* 2016, Long *et al.* 2008, Shen *et al.* 2017, Yang *et al.* 2012, Yu *et al.* 2016). The other is a duplicate gametic lethal model, interrupting two independent loci in hybrid sterility (Oka 1957, 1974), followed by *DPL1*/*DPL2* (Mizuta *et al.* 2010). Among all the hybrid sterility loci, *S5* is a major reproductive barrier regulator in cultivated rice (Ikehashi and Araki 1986, Song *et al.* 2005) and is loosely linked to *THB1* on the short arm of chromosome 6 by a physical distance of almost 5 Mb. At *S5* locus, the interaction of *S5-i* with *S5-j* alleles in sporophytes results in the abortion of female gametes that carry *S5-j* allele (Ikehashi and Araki 1986). Therefore, the selective abortion of female gametes carrying the chromosome segment of *thb1*–*S5-j* from ‘Yukihikari’ in F_1_ plants is suggested to lead to transmission ratio distortion of *THB1*–*S5-i* from *indica*. This resulted in deficient, weak F_2_ individuals in test crosses ‘Yukihikari’/‘Akamai’ (JRC21/JP9694) and ‘Yukihikari’/‘Akamai’ (JRC43/JP4744). Further studies are required to clarify whether *S5* contributes to the segregation distortion in these cross combinations. In the test cross ‘Yukihikari’/‘Touboshi’, although severe sterility was observed in F_1_ hybrids, no segregation distortion of normal plants versus weak plants were observed in F_2_ population. We need to confirm if ‘Touboshi’ and two ‘Akamai’ accessions possess different hybrid sterility loci in F_1_ hybrids with ‘Yukihikari’. In addition to female gamete abortion, hybrid sterility between *japonica* and *indica* is caused by male gamete abortion and embryo abortion (Liu *et al.* 2004). Future studies should examine the contribution of each characteristic to the variations in spikelet sterility.

### Application of dCAPS maker YK3SNP06-796157_2 in rice breeding

In the present study, we developed a codominant marker, YK3SNP06-796157_2, to determine the genotype of the causal SNP in *thb1*. The bulk population method has been widely employed in conventional rice breeding for decades (Matsubara 2020). In this method, the early generation population is subjected to natural and viable selection (Allard 1960, Ikehashi and Fujimaki 1980). Thus, many weak and/or sterile genotypes are likely to be eliminated from the population before the establishment of an advanced-generation population. A close linkage between favorable and deleterious alleles results in cosegregation (Lynch and Walsh 1998). Therefore, if a deleterious allele, *thb1*(H2) in ‘Yukihikari’, was closely linked to an allele favorable to rice breeding, the favorable allele may be eliminated in early generation. Therefore, to avoid such problems, YK3SNP06-796157_2 may be useful for selecting plants heterozygous for *THB1* in early generations. This marker-assisted selection (MAS) allows the maintenance of a closely linked favorable allele until an advanced-generation population is combined with a higher probability of breaking the linkage between *thb1*(H2) and favorable alleles. Thus, the bulk population method with MAS using YK3SNP06-796157_2 will be a challenge for rice breeding programs in the future.

Information on the distribution of hybrid breakdown-associated alleles among cross parents is a prerequisite for rice breeding (Matsubara 2020). The SNP marker developed here, YK3SNP06-796157_2, will be useful for extensively surveying cross parents. Furthermore, amplicon sequencing of *THB1* or the development of SNP arrays based on a causal SNP in *THB1* will facilitate recent genomics-assisted rice breeding.

## Author Contribution Statement

KK and TW designed the study, performed experiments, interpreted data, and wrote the manuscript. All authors have read and agreed to the final version of the manuscript.

## Supplementary Material

Supplemental Table

## Figures and Tables

**Table 1.** The spikelet fertilities of F_1_ plants and segregation analysis in F_2_ progenies derived from the crosses between testers, Kirara397 and Yukihikari, and nine accessions

Cross combination					F_1_ plants					F_2_ population					
♀	♂				Spikelt fertility (%)					No. of plants				Goodness of fit	
	Accession	SG*^a^*	HT*^b^*		2021	2022	Average	Class*^c^*		Normal	Weak	Total		χ^2^(15:1)	*p*
Kirara397	Akage (JP5301)	*tej*	H2		84.1	87.9	86.0	A		456	27	483		0.36	0.55
Kirara397	Akage (JP14994)	*tej*	H2		94.2	82.0	88.1	A		448	36	484		1.17	0.28
Kirara397	Mansaku (JRC22/JP6735)	*tej*	H3		62.4	79.6	71.0	A		463	2	465		26.88	<0.001
Kirara397	Shinyamadaho 2 (JRC37/JP6962)	*tej*	H3		87.5	83.0	85.3	A		480	1	481		29.97	<0.001
Kirara397	Sensho (JRC04/JP4386)	*trj*	H10		81.3	67.9	74.6	A		498	1	499		31.17	<0.001
Kirara397	Akamai (JRC21/JP9694)	*ind*	H22		26.8	65.3	46.1	B		449	3	452		24.07	<0.001
Kirara397	Akamai (JRC43/JP4744)	*ind*	H22		39.0	62.1	50.6	B		464	1	465		28.90	<0.001
Kirara397	Karahoushi (JRC44/JP10788)	*ind*	H22		0.4	16.7	8.6	C		492	0	492		32.80	<0.001
Kirara397	Touboshi (JRC42/JP10772)	*ind*	H22		9.2	24.6	16.9	C		464	1	465		28.90	<0.001
Yukihikari	Akage (JP5301)	*tej*	H2		60.6	78.1	69.4	A		354	1	355		21.58	<0.001
Yukihikari	Akage (JP14994)	*tej*	H2		85.1	88.9	87.0	A		457	0	457		30.47	<0.001
Yukihikari	Mansaku (JRC22/JP6735)	*tej*	H3		77.3	97.2	87.3	A		427	33	460		0.67	0.41
Yukihikari	Shinyamadaho 2 (JRC37/JP6962)	*tej*	H3		76.9	73.1	75.0	A		415	28	443		0.00	0.95
Yukihikari	Sensho (JRC04/JP4386)	*trj*	H10		81.0	86.9	84.0	A		378	24	402		0.05	0.82
Yukihikari	Akamai (JRC21/JP9694)	*ind*	H22		7.5	27.2	17.4	C		408	15	423		5.28	0.02
Yukihikari	Akamai (JRC43/JP4744)	*ind*	H22		2.1	28.6	15.4	C		415	15	430		5.60	0.02
Yukihikari	Karahoushi (JRC44/JP10788)	*ind*	H22		2.4	1.2	1.8	C		162	1	163		8.84	<0.001
Yukihikari	Touboshi (JRC42/JP10772)	*ind*	H22		6.3	8.8	7.6	C		272	18	290		0.00	0.98

*^a^* Subgroup, *tej*: *temperate japonica*, *trj*: *tropical japonica*, *ind*: *indica*. *^b^* Haplotype at *THB1* region. *^c^* A: more than 61%, B: more than 21% and less than 60%, C: less than 20%.
