# Supplementary material for: THB1, a putative transmembrane protein that causes hybrid breakdown in rice
Source: Breed Sci. 2024 Jun 13;74(3):193–203. doi: 10.1270/jsbbs.23065 (PMC11561410; doi:10.1270/jsbbs.23065)
Supplement: Supplementary file 1 — Supplemental Table [file 74_193-s1.pdf]

doi: 10.1270/jsbbs.23065

Supplemental Table 1 Characteristics of the CAPS and dCAPS markers developed in the present study

Marker name	Type	Position[a] (bp)	Primer sequence (5 --> 3')	AT[b] (°C)	PCR product size (bp)	RE[c]	Restriction fragment length (bp) Yukihikari/Kirara397
YK3SNP06-660026	CAPS	660,125	AACTGCTCATTCCTCCACCA<br>GCATACCTGAACGTTGGCAT	60	224	*Mae* I	224/117+53
YK3SNP06-710081	dCAPS	711,080	GGTATACAGTTAAACAAATAGTCTG<br>CGTGGTTTCAGCACACAGAA	53	237	*Pst* I	210+27/237
YK3SNP06-728703	dCAPS	729,702	CTTGTGTACACTTGCATTTTAAAAC<br>ATCTCGTCCTTTCCCAACCT	53	106	*Mae* I	81+25/106
YK3SNP06-760888	dCAPS	761,887	TGGGAAGTCATCAAGCAACAAATTC<br>CCCCTGCACTACTCCTTTCA	53	249	*Taq* I	224+25/249
YK3SNP06-792528	dCAPS	793,527	ATAAATAAATCTACAAGTAATATTA<br>TGGCCATGAGTTATTTTCCA	53	211	*Sca* I	187+24/211
YK3SNP06-796157_2	dCAPS	797,156	CAGCTGCATGCGAACAGCATCAGTA<br>CCACAATGTGCATCTTCACC	53	173	*Rsa* I	173/148+25
YK3SNP06-801669	dCAPS	802,668	TTCTCTGCTGTACATTTTTTTTCTG<br>ACCTTACCCCCATTCAGTCC	53	184	*Pst* I	184/166+18
YK3SNP06-834006	dCAPS	833,005	GGGAACGATGAGCTGTCTCGGTCCC<br>CGGTCTTTCAAATTTTGGTCA	53	155	*Mae* I	131+24/155
YK3SNP06-837721	dCAPS	838,720	ATGGTGAAGAAATCTCCTGTCATCC<br>CACGTAAGAACATGCAAACAGC	60	107	*Mae* I	107/82+25

[a] Physical position (bp) on chromosome 6 (Nipponbare IRGSP-1.0 reference genome)

[b] Annealing temperature

[c] Restriction enzyme
